# Utilization of non-Ebola health care services during Ebola outbreaks: a systematic review and meta-analysis

**DOI:** 10.7189/jogh.09.010406

**Published:** 2019-06

**Authors:** Jess Alan Wilhelm, Stéphane Helleringer

**Affiliations:** 1 Johns Hopkins University, Bloomberg School of Public Health, Department of International Health, Baltimore, Maryland, USA; 2 Johns Hopkins University, Bloomberg School of Public Health, Deptartment of Population, Family, and Reproductive Health, Baltimore, Maryland, USA

## Abstract

**Background:**

Beyond their direct effects on mortality, outbreaks of Ebola Virus Disease (EVD) might disrupt the provision of health care services in affected countries, possibly resulting in an increase in the number of deaths from non-EVD causes. We conducted a systematic review and meta-analysis of studies documenting the impact of EVD outbreaks on health care utilization.

**Methods:**

We searched PubMed, Embase, Scopus, Web of Science, Cumulative Index to Nursing and Allied Health Literature (CINAHL), Global Health, Pascal and grey literature to identify observational studies that compared indicators of health care utilization before and during the outbreak. We identified 14 752 unique citations, 22 of which met inclusion criteria. All were related to the 2013-2016 West African EVD outbreak. From the 22 studies, we extracted 235 estimates of the relative change in health care utilization during the EVD outbreak. We used multivariate regression to estimate the average effect of the outbreak on health care utilization, and to assess heterogeneity across study characteristics.

**Findings:**

On average, health care utilization declined by 18.0% during the outbreak (95% Confidence Interval: -26.5%, -9.5%). The observed declines in health care utilization were largest in settings affected by higher levels of EVD incidence (>2.5 cases per 100 000 per week) whereas utilization did not change in settings with EVD incidence less than 0.5 cases per 100 000 per week. Declines in utilization were greater for inpatient care and for deliveries than for outpatient care. They were also larger in studies based on small samples of health facilities, suggestive of publication bias. However, several studies based on larger samples of facilities also observed declines in health care utilization.

**Conclusions:**

During the West African EVD outbreak, the utilization of health services declined significantly. During outbreaks of EVD, attention needs to be paid to the disruption of the health services, which can have large indirect health impacts.

The 2013-2016 Ebola Virus Disease (EVD) outbreak in West Africa was responsible for 28 616 cases and 11 310 deaths [1]. There are, however, strong concerns that the outbreak may have precipitated an additional number of deaths through its indirect effects [2-7]. These include, for example, deaths resulting from increased malnutrition and poverty in a context of reduced economic activity. The World Bank estimates the loss of GDP in Guinea, Liberia, and Sierra Leone due to Ebola at $2.8 billion USD through 2015 [8]. Additional deaths may also have resulted from a sharp reduction in the coverage of essential health interventions during the course of the outbreak.

Healthcare utilization, which we define as the use of facility-based formal health services, may indeed have declined for several reasons. First, the outbreak may have affected the supply of health services because health workers experienced a particularly heavy death toll; by June 2015, 515 health care workers had died [9]. Since there were already few health workers per capita in the most affected countries, this likely significantly reduced the supply of health services. In some settings, this might even have precipitated the closure of some health facilities, or some selected services within facilities. On the other hand, the remaining financial and human resources were likely diverted away from the provision of regular health services and re-allocated to the Ebola response. Finally, some health workers may also have discontinued engaging in certain procedures that posed high risk of infection with EVD (eg, C-sections).

Second, the demand for health services may also have been affected. Potential patients may have avoided seeking care at health facilities because they feared contracting or being diagnosed with EVD during their visit. In some settings, concerns about the potential for nosocomial EVD transmission [10] led to beliefs that health facilities should be avoided. Similar effects have already been documented in prior outbreaks of other infectious diseases, eg, the 2003 SARS outbreak, which was associated with a decline in health care expenditures for inpatient and ambulatory care in Taiwan [11].

These dynamics of the supply and demand of health care during an EVD outbreak might have led to an additional number of non-EVD deaths if a) healthy individuals could not access preventive services, and b) sick individuals could not obtain required life-saving treatments. Early in the West African EVD outbreak, several studies thus projected that thousands of additional deaths from measles [2,3] and malaria [4] may result from the effect of the EVD outbreak on health care services, while others have pointed towards disruption of care for HIV [5] and reproductive health [6]. Unfortunately, the extent of this indirect death toll has not been measured due to limited data on the number of deaths due to non-EVD causes that occurred both before and during the outbreak [12].

Instead, several studies have documented changes in the utilization of health care services during the outbreak. In this paper, we attempt to summarize the evidence provided by these studies through a systematic review and meta-analysis. In doing so, we improve on a prior summary of this literature, conducted by Brolin Ribacke et al. (2016) [7] in several ways. First, we include eight additional articles published in 2016, as well as several reports obtained from national ministries and non-governmental organizations. Second, we only include studies with empirical data collected before/during and possibly after the outbreak. We exclude studies built on simulations, which may reflect the assumptions of the modeling team rather than the true effects of EVD on health care utilization. Finally, we conduct a meta-analysis of the findings.

## METHODS

We searched PubMed, Embase, Scopus, Web of Science, Cumulative Index to Nursing and Allied Health Literature (CINAHL), Global Health, and Pascal. While databases were initially searched between September and December of 2016, all final searches were repeated on December 22, 2016. We searched all articles since the discovery of EVD in 1976 [13]. The search vocabulary for PubMed (Box 1) was adapted for use with each database. All search terms were in English, except for French-language terms used in Pascal. No language restrictions were used in the literature review provided the articles could be identified using English and French-language search terms. We did not register this systematic review, but we have followed the PRISMA guidelines (see Table S1 in the **Online Supplementary Document**). Since our study does not investigate the effect of an intervention, but rather evaluates the impact of a temporal exposure through observational data, various items of the checklist are not applicable. For example, detailed information on the participants is not reported consistently as it would be in a clinical trial.

Box 1 **PubMed search strategy**

(“Hemorrhagic Fever, Ebola”[Mesh] OR ebola[tw])

AND

((“Health Services Accessibility”[Mesh] OR “Delivery of Health Care”[Mesh] OR “Quality Assurance, Health Care”[Mesh] OR “Quality of Health Care”[Mesh] OR “Hospitalization”[Mesh] OR “Ambulatory Care”[Mesh] OR effect*[tw] OR impact*[tw] OR utilization[tw] OR utilisation[tw] OR performance[tw]) OR (“Obstetrics”[Mesh] OR “Pregnancy”[Mesh] OR “Malaria”[Mesh] OR “Child Health Services”[Mesh] OR “Maternal Health Services”[Mesh] OR “Immunization”[Mesh] OR “Vaccination”[Mesh] OR “Immunization Programs”[Mesh] OR “Mass Vaccination”[Mesh] OR “HIV Infections”[Mesh] OR “Tuberculosis”[Mesh] OR “Anti-Retroviral Agents”[Mesh] OR “Heart Diseases”[Mesh] OR “Vascular Diseases”[Mesh] OR “Diabetes Mellitus”[Mesh] OR health care[tw] OR health service*[tw] OR inpatient[tw] OR outpatient[tw] OR obstetric*[tw] OR pregnant[tw] OR pregnanc*[tw] OR malaria[tw] OR “child health”[tw] OR “maternal health”[tw] OR vaccinat*[tw] OR immuniz[tw] OR immunis*[tw] OR “HIV”[tw] OR “PMTCT”[tw] OR “tuberculosis”[tw] OR “non-communicable diseases”[tw] OR heart disease*[tw] OR diabetes[tw] OR “antiretroviral treatment”[tw] OR indirect[tw] OR secondary[tw]))

The seven databases we searched yielded 25 788 citations. We used EndNote to conduct de-duplication of records, using the method outlined by Bramer et al. (2016) [14]. This resulted in a total of 14 741 unique citations. In addition, we added 11 articles from a) a grey literature search using Google Scholar (6 articles), b) forward searches of citations within articles (2 articles), c) suggestions from experts (3 articles). In total, 14 752 unique citations were identified.

**Figure 1:**
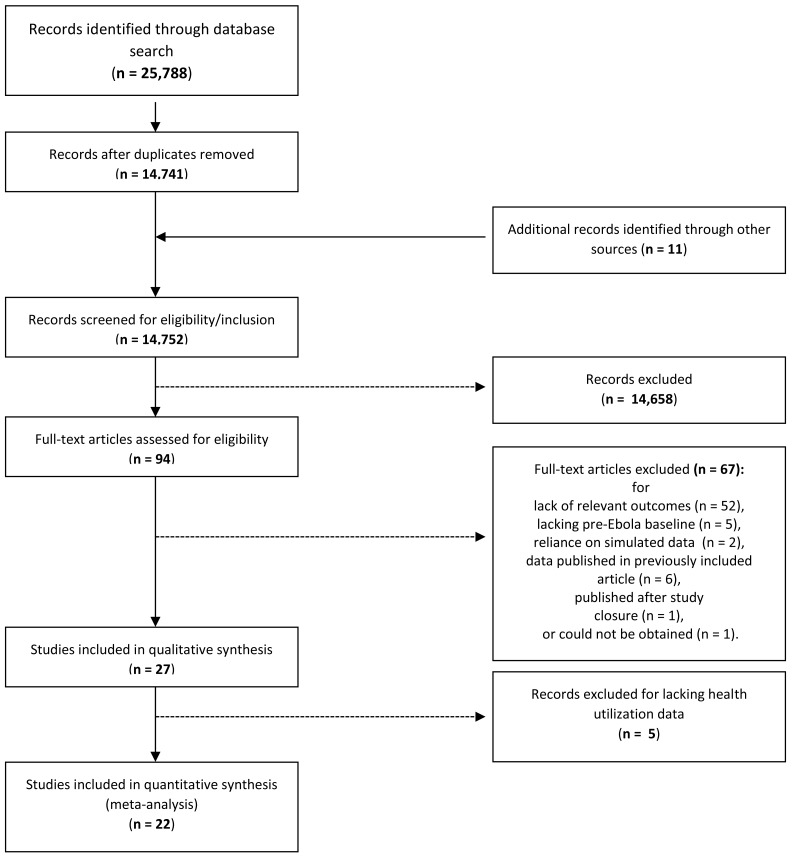
Literature review flowchart.

Two reviewers independently assessed the titles and abstracts for relevance and likelihood of meeting the inclusion criteria. Articles were excluded if 1) they did not contain quantitative empirical data documenting outcomes during an EVD outbreak and instead relied solely on simulations, mathematical models or qualitative data; 2) did not include comparison data from a pre-Ebola period or from a population not exposed to an EVD outbreak; or 3) did not include a health care utilization outcome other than EVD-related outcomes. One author, JW, supervised the title-abstract review process. The first 3000 citations were independently screened by JW and feedback was provided to reviewers. JW screened an additional sample of 3500 articles to ensure that all potentially relevant citations were included in full-text screening. JW did not identify any relevant citations missed by the reviewers. The title-abstract review identified 94 citations. Full-text articles were then extracted and assessed for inclusion by JW. A total of 26 relevant articles were identified. Reasons for exclusion at this stage included primarily lack of data on relevant outcomes, lack of a pre-EVD outbreak baseline measurement, and reliance on simulated data. Even though our search potentially included articles describing early EVD outbreaks (eg, the 2000 outbreak in Uganda), all remaining articles described studies conducted during the 2013-2016 West African EVD outbreak. One article recommended by an expert was excluded for being published after study closure. **Figure 1** contains the flowchart describing these inclusions/exclusions according to PRISMA guidelines.

Two reviewers independently extracted data from the 26 articles using a standardized template, including information on study setting, design, analysis methodology, reported results, and limitations. At this time, reviewers also assessed study quality and recorded unreported study limitations. Study quality assessments were made by evaluating the methodology (pre-post, longitudinal cohort, cross-sectional) sampling methods (census, random, convenience), types of biases present (eg, recall, non-response), extent of missing data, number of facilities and duration of the Ebola period studied, and control for confounders, including seasonality. JW reconciled differences between the two reviews.

### Meta-Analysis Methods

We restricted our meta-analysis to 22 articles (identified in **Table 1**) [6,15-35] that contained information on non-EVD health care utilization. We excluded 4 studies [36-39] that contained only data on non-Ebola health outcomes, such as all-cause mortality. **Table 1** presents key information for the 22 articles included in the meta-analysis. Each study potentially provided multiple estimates of the impact of EVD on health care (eg, if it included data from multiple districts and/or data on several types of services).

**Table 1:** Summary of articles with utilization outcomes

Source	Country	Location	Study Design	Outcomes	Pre-Ebola Period (Number of Months)	Ebola Period (Number of Months)	Control for Seasonality	Number of Health Centers, Hospitals (Total)	Sampling	Limitations	Sources of Funding	Notable Author Affiliations	Study Quality	Risk of Bias
**Ortuno-Gutierrez et al. (2016)**	Guinea	Conakry	Pre-Post	HIV, TB	Apr-Dec 2013 (9 mo)	Apr-Dec 2014 (9 mo)	Yes	1, 0 (1)	None	Single facility, had an intervention to maintain quality of care during Ebola	Damien Foundation	Medecins Sans Frontieres (MSF) / Doctors Without Borders;National Ministry of Health	Good	High
**Moisan et al. (2016)**	Guinea	Guèckèdou	Pre-Post	Malaria, OPD, Other Child Diseases	Malaria: April-July 2012 & 2013 (4 mo); OPD: Apr-Sep 2013 (6 mo); ARI: Sept-Dec 2013 (4 mo)	Malaria: April-July 2014 (4 mo); OPD: Apr-Sep 2014 (6 mo); ARI: Sept-Dec 2014 (4 mo)	Yes	38, 0, (38)	None	Missing data likely, but not reported		National Ministry of Health	Good	Medium
**Leuenberger et al. (2015)**	Guinea	Macenta	Pre-Post	HIV, OPD, TB	Aug - Dec 2013 (5 mo)	Aug - Dec 2014 (5 mo)	Yes	0, 1 (1)	None	Single facility	[US] National Institutes of Health		Fair	High
**Barden-O'Fallon, Barry, Brodish, Hazerjian (2015)**	Guinea	Nationwide (Sampled: Guèckèdou, Conakry, Boffa, Coyah, Dabola, Dalaba, Faranah, Fria, Kissidougou, N’Zèrèkorè, Siguiri, Mamou and Mandiana)	Pre-Post	ANC, FBD, FP, HIV, Imm, OPD, Other Child Diseases	Oct-Dec 2013 (3 mo)	Oct-Dec 2014 (3 mo)	Yes	29, 16 (45)	Convenience	Used a purposive sampling method; Large amount of missing data for HIV outcomes	USAID		Fair	Medium
**Plucinski, 2015**	Guinea	Nationwide (Sampled: Conakry, Fria, Gaoual, Labe, Guèckèdou, Macenta, Kerouane, Mandiana)	Difference-in-Difference	ANC, Malaria, OPD	Jan - Nov 2013 (11 mo), April-July 2013(4 mo), Aug-Nov 2013 (4 mo)	Jan - Nov 2014 (11 mo), April-July 2014 (4 mo), Aug-Nov 2014 (4 mo)	Yes	112, 8 (120)	Probability	Did not adjust for survey design; did not calculate confidence intervals	Global Fund to Fight AIDS, Tuberculosis and Malaria, and President’s Malaria Initiative	US Centers for Disease Control (CDC), Catholic Relief Services, National Ministry of Health	Fair	Low
**Ndawinz et al. (2015)**	Guinea	Conakry	Pre-Post	HIV	Jun-14 (1 mo)	November 2014 (1 mo)	No	0, 1 (1)	None	Single hospital	Solidarité Thérapeutique et Initiatives pour la Santé	National Ministry of Health	Fair	High
**Delamou et al. (2014)**	Guinea	Conakry	Pre-Post	Inpatient	July-September 2013 (3 mo)	July-September 2014 (3 mo)	Yes	0, 1 (1)	None	Single facility, short period, limited information		National Ministry of Health, MSF	Fair	High
**Ly et al. (2016)**	Liberia	Rivercess	Cross-sectional Survey with Recall	FBD	March 2011 - June 14, 2014 (39.5 mo)	June 15, 2014 - April 13, 2015 (10 mo)	No	N/A	Probability	Social desirability and recall bias	Direct Relief and UBS foundation	Last Mile Health; National Ministry of Health	Excellent	Low
**Loubet et al. (2015)**	Liberia	Monrovia	Time-series	HIV	Jan-May 2014 (5 mo)	June-Oct 2014 (5 mo)	Yes	0, 2 (2)	Convenience	Few facilities	Ensemble pour une Solidarite Therapeutique en Reseau (ESTHER) France provided funding for the electronic patient database system	National Ministry of Health	Good	High
**Lori et al. (2015)**	Liberia	Bong	Pre-Post	FBD	Jan-July 2014 (7 mo)	Aug-Oct 2014 (3 mo)	No	10, 2 (12)	Convenience	Small, purposive sample	USAID; Fogarty International; National Institutes of Health (NIH)		Fair	Medium
**Iyengar, Kerber, Howe, & Dahn (2015)**	Liberia	Margibi, Bong	Pre-Post	ANC, FBD, Malaria	March 2014 (1 mo)	August or September 2014 (1 mo)	No	Not Reported	None	Limited data on facilities; May be affected by reporting	Save the Children (Saving Newborn Lives program)	Save the Children, National Ministry of Health	Fair	High
**Tattevin et al. (2015)**	Liberia	Monrovia	Pre-Post	HIV	Jan-May 2014 (5 mo)	June-Oct 2014 (5 mo)	No	0, 1 (1)	None	Single facility; Short Ebola Period		National Ministry of Health	Fair	High
**Liberia MoHSW (2014)**	Liberia	Nationwide (with ANC by county)	Pre-Post	ANC, FBD, FP, HIV, Imm, OPD, Malaria, Other Child Diseases, TB	Jan - Dec 2013 (12 mo)	Jan - Dec 2014 (12 mo)	Yes	651, 36 (687)	None	Missing data (reporting rate declined 83% in 2013 to 72% in 2014). Excludes private facilities.		National Ministry of Health	Fair	High
**Grépin and Chunara (2015)**	Liberia	Nationwide	Cross-sectional Survey with Recall	FBD	March 2014 (1 mo)	September 2014 (1 mo)	No	N/A	Convenience (SMS-based sample)	Recall and social desirability bias. Did not present evidence that the SMS sample was nationally representative.	UK Aid		Fair	Medium
**Brolin Ribacke et al. (2016)**	Sierra Leone	Nationwide	Pre-Post	Inpatient, FBD	Jan - May 2014 (5 mo)	FBD: May-Dec 2014 (8 mo), C-sections/FBD: June-Dec 2014 (7 mo), Jan-May 2015 (5 mo)	Yes (Jan-May 2015), No (May/June-Dec 2014)	0, 32 (32)	None	Missing data imputed to zero	Swedish National Board of Health and Welfare; The Wallenburg Foundation; CapaCare; Norwegian University of Science and Technology	National Ministry of Health	Good	Low
**Bundu et al. (2016)**	Sierra Leone	Freetown	Pre-Post	Surgery, Inpatient	Surg: Aug 2013 (1 mo); INP: Dec 2013 (1 mo)	Surg: Aug 2014 (1 mo), INP: December 2014 (1 mo)	Yes	0, 1 (1)	None	Single Hospital	King's Sierra Leone Partnership, King's Centre for Global Health	National Ministry of Health	Fair	High
**Elston et al. (2015)**	Sierra Leone	Moyamba; Koinadugu	Pre-Post	FBD, Imm, Inpatient, OPD	Aug-Dec 2013 (5 mo), Oct-Jan 2013 (4 mo), Jan-June 2014 (6 mo)	Aug-Dec 2014 (5 mo), Oct-Jan 2014 (4 mo), July-Dec 2014 (6 mo)	Yes, No (Imm)	4, 1 (5)	Convenience	Few facilities, selective reporting of outcomes		National Ministry of Health; Doctors of the World	Fair	High
**Bolkan et al. (2014)**	Sierra Leone	Nationwide	Pre-Post	Inpatient, Surgery	Jan - May 2013 (5 mo)	June-Oct 2014 (5 mo)	No	0, 40 (40)	None	Missing data	CapaCare, Norwegian University of Science and Technology, national board of health and welfare in Sweden	National Ministry of Health	Fair	Low
**Jones et al. (2016)**	Sierra Leone	Nationwide	Pre-Post	ANC, FBD	April 2013 - Mar/Apr 2014 (12 to 13 mo)	April/May 2014 - Jan 2015 (9 to 10 mo)	Yes	65, 13 (78)	None	Missing data on ANC/PNC, Limited to EMoC facilities	WaterAid - grant from Voluntary Service Overseas		Good	Low
**UNICEF Sierra Leone Health Facility Survey (2015)**	Sierra Leone	Nationwide	Pre-Post	ANC, FBD, HIV, Imm, Malaria, Malnutrition	May 2014 (1 mo)	September 2014 (1 mo)	No	1137, 0 (1137)	None	Extent of missing not reported		UNICEF, National Ministry of Health	Good	Medium
**UNFPA Rapid Assessment (2015)**	Sierra Leone	Nationwide	Pre-Post	ANC, FBD, FP, Imm	Jan 2013 - Apr 2014 (16 mo)	May-Sept 2014 (5 mo)	Yes	Not Reported	None	Limited data on reporting facilities			Fair	Medium
**Quaglio et al. (2016)**	Sierra Leone	Pujehun	Pre-Post	FBD, Inpatient, Surgery	FBD/INP: July-Dec 2013 (6 mo), Jan-Dec 2013 (12 mo)	FBD/INP: July-Dec 2014 (6 mo), Jan-Dec 2014 (12 mo)	Yes	0, 1 (1)	None	Single hospital	Doctors with Africa (DwA) CUAMM	National Ministry of Health	Fair	High

ANC – antenatal or postnatal care, FBD – facility-based deliveries, FP – family planning, HIV – HIV service delivery, Im – immunizations, Inpatient – impatient admissions including maternity, Malaria – diagnosis or treatment of malaria, Malnutrition – diagnosis or treatment of child or maternal malnutrition, OPD – outpatient department visits, Surgery – inc. C-section, TB – tuberculosis services, mo – months

From each of these papers, we extracted reported counts of patients/clients or coverage contained in tables, text, and/or figures. We classified the data by time period (before vs during the outbreak) and we calculated the relative change between the two periods. One study of facility-based deliveries [26] reported an odds ratio of 0.69 associated with the EVD outbreak, which we converted to a change of -31%. Although this is not a valid data interpretation without information on prevalence, we deemed it better to include the data point with some risk of bias than to exclude it. On the other hand, one estimate of trends in HIV services from a segmented linear regression model at a single hospital in Liberia could not be converted into a percent change [25].

For each estimate of the relative change in utilization, we recorded study metadata including service type (eg, HIV treatment, family planning delivery), time period during Ebola outbreak (eg, early vs late), study location, sampling strategy used, controls for seasonal variation, data source (eg, health information system or ancillary survey), and number and level of health facilities in the sample. The time period of the Ebola outbreak is that for which we were best able to extract a percent change estimate of utilization from the article, which is not always the entire observation period studied by the original study. For example, Iyengar et al. (2015) track utilization from March to December 2014, but only report declines numerically for August relative to March. To control for the bias introduced in this way, we adjust for the duration of the Ebola period (in months) and the intensity of transmission (mean incidence during the period) in the meta-regression model.

In order to identify the level of EVD incidence during the time of the study, we matched each study location and time period with data on EVD cases from Backer and Wallinga (2016) [40]. We considered using peak incidence and cumulative incidence as measures of exposure to EVD under the assumption that health care utilization may be particularly responsive to a high number of EVD cases occurring in a short period of time. Ultimately, we use mean incidence rather than peak incidence, because the mode is subject to much more noise than the mean for rare events. We also considered using the number of past EVD cases in a location as our measure of exposure. However, most reported estimates of the impact of EVD on non-EVD health care services were based on the EVD period from the beginning of the outbreak, thus making cumulative and average incidence identical.

In total, we obtained 235 estimates of the change in health care utilization during the course of the EVD outbreak (mean = 10.7 estimates per study, standard deviation = 21.5). Most studies reviewed did not report confidence intervals. This was so in large part because they relied on data from health information systems, which include complete counts/reports of the number of patients attending various health services for facilities that report (see **Table 1**). As a result, we could not perform conventional meta-analysis using inverse-variance weighting [41]. Instead, we tested for differences in health care utilization between the pre-outbreak period and the period during the outbreak as follows. First, we used Student’s *t* test to detect differences in the mean level of utilization between the “before” and “during” periods.

Second, suspecting non-normal distributions of study effects, we used the non-parametric, cluster-adjusted Wilcoxon signed rank test to detect differences in the median level of utilization between the two periods. Finally, we used ordinary least squares (OLS) regressions, in which the level of health care utilization was the outcome, and covariates included a dummy variable identifying the time period covering the EVD outbreak, as well as the metadata described above. We used OLS to identify potential sources of heterogeneity in the effects of the outbreak on health care utilization, with adjustment for clustering by study. We used a Wald-test to identify effect measure modification by sources of heterogeneity. The percent change was normally distributed, except at the extreme-right side. Regressions with log-transformation of the dependent variable to account for right-skew did not alter the conclusions. All meta-analysis methods were identified post hoc, after the studies had been reviewed. Data management and analysis were performed in R version 3.4.3 [42] and meta-regression analyses were conducted in Stata 15 [43].

## RESULTS

### Summary of evidence

**Figure 2:**
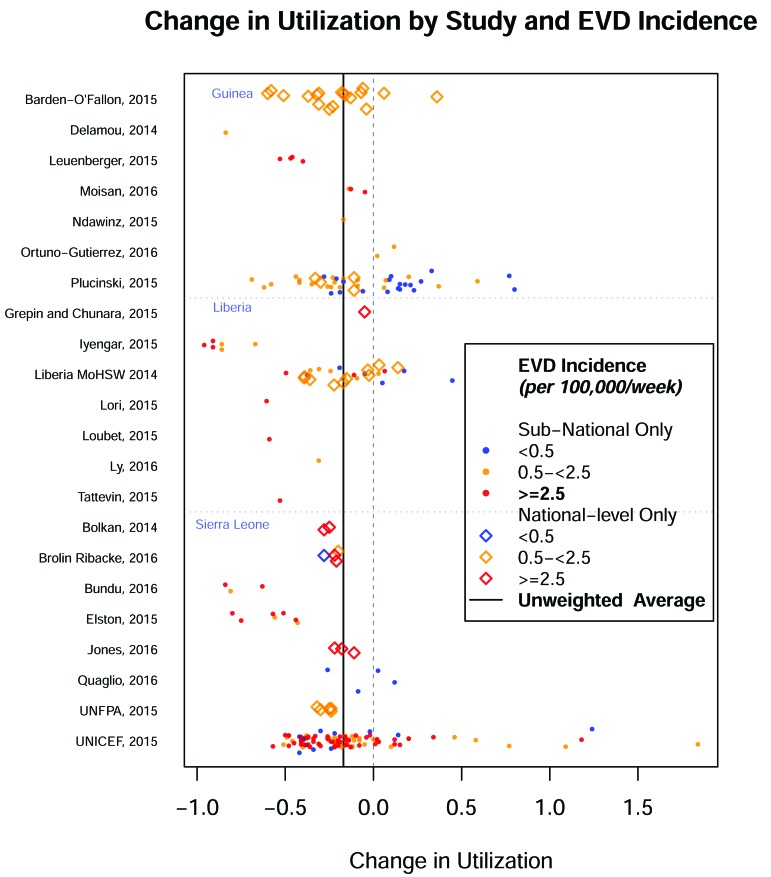
Change in utilization by study and Ebola incidence.

**Figure 2** presents the estimates by study and level of EVD incidence. One outlier representing a 500% increase from a low baseline [15] was dropped from the subsequent analysis. National-level data are available only for 48 estimates and sub-national data are available for estimates 188 estimates. If we aggregate complete sub-national data to the national-level, we can have 65 national-level estimates.

Of the estimates, 28 relate to facility-based deliveries (FBD), 41 to antenatal or post-natal care (ANC), 15 to outpatient department visits (OPD), 15 to forms of inpatient care (inpatient admissions, surgeries & C-sections) other than vaginal facility-based deliveries, 50 to malaria (treatment, prevention, and diagnostics) in an outpatient setting, 27 to HIV or TB-specific services, 20 to immunizations, and 39 to other services (eg, malnutrition, diarrhea).

The mean incidence of EVD during the observation period for which the effect estimate can be extracted varied greatly. Eighty utilization estimates (34%) were derived from a setting with mean Ebola incidence of ≥2.5/100 000 population/week, 111 (47%) from settings with 0.5 to 2.5/100 000 /week, and 44 (19%) were associated with a mean incidence of Ebola of <0.5/100 000 /week, including 20 (8%) reported from locations inside of the affected countries that were free of Ebola during the study period.

**Figure 3:**
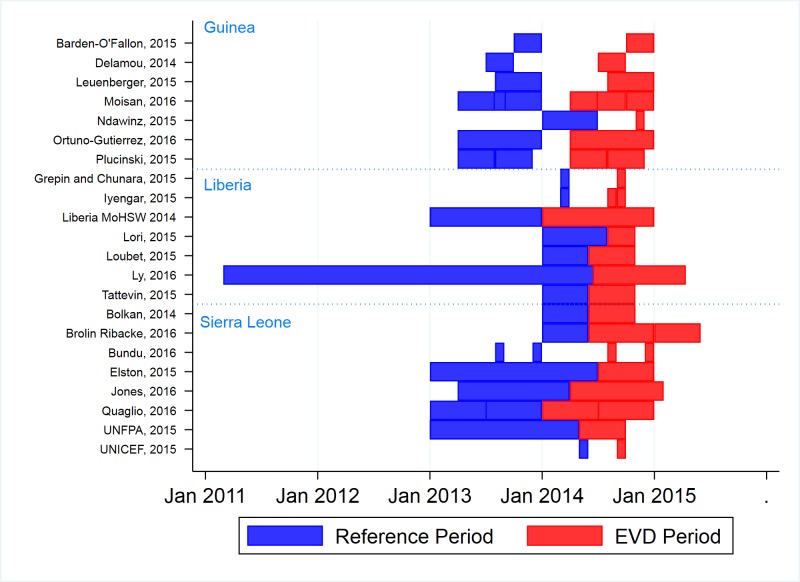
Distribution of study reference and Ebola exposure periods (2011-2015).

The mean duration of the Ebola period for estimates was 3.65 months (median: 3 months, IQR: 1-4 months). Ebola periods reported for estimates varied from Jan 2014 to May 2015 (**Figure 3**).; however, most estimates of utilization during the Ebola period come from April – December 2014, which contains peak transmission period for the three countries that occurred between September and December 2014 [44]. Baseline periods varied, but mostly fell between early 2013 and early 2014.

### Meta-Analysis Results

**Table 2** presents the mean and median change in utilization during the Ebola period for all health services. The mean change in utilization was -18.0% (95% confidence interval: -26.5%, -9.5%) and the median was -21.0% (IQR: -37.8%, -4.9%). There was an apparent dose-response relationship with the local EVD incidence rate, with a change in health care utilization of only -0.5% in areas where the EVD incidence was less than 0.5 cases per 100 000/week, vs -18.0% and -27.6% in areas where the EVD incidence was between 0.5 and 22.5 cases per 100 000/week and greater than 2.5 cases 100 000/week, respectively. There was no significant change in mean utilization of health care services in the lowest EVD incidence category for any type of service. On the other hand, large declines were seen for EVD categories above 0.5/100,000/week for every service.

**Table 2:** Changes in utilization for all service types

Level	Mean EVD incidence (cases per 100 000 pop. per week)	N	Cluster-adjusted mean proportion change (robust 95% CI)	Unweighted mean change	T-test p-value (H0 = 0)	Median change (inter quartile range)	Cluster-adjusted Wilcoxon signed rank test *P*-value
**All**	All	235	**-0.180 (-0.265, -0.095)**		<0.001	**-0.210 (-0.378, -0.049**)	<0.001
	<0.5	44		-0.005	0.918	-0.054 (-0.245, 0.150)	0.347
	0.5-<2.5	111		**-0.180**	<0.001	**-0.210 (-0.359, -0.085**)	0.004
	≥2.5	80		**-0.276**	<0.001	**-0.255 (-0.453, -0.130**)	0.001
**National-level & Aggregated**	All	65	**-0.170 (-0.230, -0.110)**		<0.001	**-0.210 (-0.292,-0.083**)	0.019
	<0.5	11		-0.055	0.494	-0.110 (-0.285, 0.087)	0.162
	0.5-<2.5	39		**-0.205**	<0.001	-0.223 (-0.315, -0.110)	0.071
	≥2.5	15		**-0.161**	0.005	-0.220 (-0.240, -0.145)	0.058
**Sub-national only**	All	188	**-0.174 (-0.278, -0.069)**		0.003	**-0.210 (-0.400, -0.015**)	0.002
	<0.5	43		0.001	0.986	-0.049 (-0.240, 0.150)	0.600
	0.5-<2.5	73		**-0.166**	0.001	**-0.210 (-0.400,-0.080**)	0.008
	≥2.5	72		**-0.286**	<0.001	**-0.305 (-0.473,-0.130**)	0.004

EVD – Ebola virus disease, CI – confidence interval

Inpatient care exhibited the largest decline during the EVD outbreak (mean change of -44.3%, *P* < 0.001; median: -36.0%, *P* < 0.019) followed by facility-based deliveries (mean: -27.7%, *P* < 0.001; median: -22.5%, *P* = 0.004). Malaria services had the smallest change, but it was still -18.2% (*P* = 0.001, median: -24.0%, *P* = 0.112). One subset of services among the “other” category that actually increased by 22% during the outbreak was treatment of child malnutrition, but this estimate was based solely on data from one study conducted in Liberia.

**Table 3** presents the results of multivariate regression models. The difference between the highest and lowest EVD categories is significant with conventional standard errors, but not after controlling for clustering of estimates within studies (-21.4 percentage points, *P* = 0.08). A Wald test for effect measure modification by EVD incidence is still statistically significant (*P* = 0.007). Declines in health care utilization were larger for inpatient services & facility-based deliveries than for outpatient services (-10.5 percentage points, *P* = 0.004). Several features of the study design were associated with the magnitude of estimates. For example, estimates derived from a single facility or with no data on the number of facilities had significantly larger declines in utilization than those with 51 or more health facilities included. A Wald test for number of facilities is significant (*P* < 0.001). Estimates for EVD periods *greater than 3 months* had less negative changes in utilization, but this difference was not significant.

**Table 3:** Multivariate regression of change in utilization

		Model I: conventional standard errors		Model II: cluster-robust standard errors	
		**Beta**	***P*-value**	**Beta**	***P* -value**
**Intercept**		0.029	0.639	0.029	0.726
**Mean Ebola intensity (per 100 000 per week)**	<0.5	Ref		Ref	
	0.5-2.5	**-0.124**	0.042	-0.124	0.287
	≥2.5	**-0.214**	0.003	-0.214	0.081
**Service type**	Inpatient & FBD	-0.105	0.089	**-0.105**	0.004
	Outpatient	Ref		Ref	
**Country**	Liberia	-0.137	0.069	-0.137	0.255
	Guinea	-0.056	0.463	-0.056	0.528
	Sierra Leone	Ref		Ref	
**Number of facilities**	Single or no data	**-0.285**	<0.001	**-0.285**	0.002
	2-20	-0.190	0.091	**-0.190**	0.006
	21-50	-0.041	0.516	-0.041	0.473
	≥51	Ref		Ref	
**Number of months**	≤3 months	Ref		Ref	
	3 to 12 months	0.098	0.188	0.098	0.212
**Control for seasonality**	Yes	-0.008	0.935	-0.008	0.930
**Sampling**	Convenience	-0.009	0.921	-0.009	0.941
	Census or probability	Ref		Ref	
**R^2^**		0.188		0.198	
**N**		235		235	

FBD – facility-based deliveries

## DISCUSSION

This review and meta-analysis provides strong evidence that health care utilization declined substantially during the early and peak phases of the 2013-2016 Ebola outbreak in West Africa. Furthermore, the magnitude of the decline in utilization was associated with local EVD incidence, and remained in studies that controlled for background seasonal variation in health care utilization. These findings corroborate the likely existence of a causal link between the occurrence of the EVD outbreak and the concomitant changes in health care utilization. We also found that utilization declined across all service types, but some services were more resilient than others. In particular, inpatient services, including facility deliveries, were more severely affected than outpatient services. Encouragingly, the estimated change in utilization in areas that had <0.5/100,000/week Ebola incidence was only -0.5%. This suggests that the effects of Ebola on health care utilization did not spillover to areas with little to no Ebola. However, it should be noted that levels of utilization in the three countries were inadequate before the outbreak.

This systematic review and meta-analysis has a number of limitations. Foremost, we are limited to studies that have been published, and there is evidence suggestive of publication bias. Estimates derived from small numbers of facilities or shorter periods tend to have larger declines. However, this potential bias does not entirely explain the observed decline in utilization. For studies with more than 50 facilities, the change in utilization is still -9.5% with a *t* test p-value of 0.003. Second, nearly all studies were based on pre-post designs that did not account for secular trends in health care utilization. This would have required a control group that was not exposed to the EVD outbreak, along with the use of difference-in-differences estimates. The absence of a control group to model the counterfactual could bias our estimate of the effect of EVD on utilization. Reviewed studies mostly address the situation in late 2014, at the peak of the EVD outbreak, and we cannot infer the effects during the latter part of the outbreak. Delamou et al. (2017) [45] and Bienvenu et al. (2017) [46], both published too late to be included in this review, found that number of ANC visits and facility-based deliveries in the forest region of Guinea had not recovered to pre-Ebola levels as late as July of 2016.

Other limitations were introduced by the methodology used in this literature review. Lacking data on facility catchment areas, we do not weight estimates to account for the population affected, nor do we control for the non-random distribution of study locations across the three countries. Double counting of data for the same services at the same facilities during the same periods does occur, which violates the assumption of independent errors and results in standard errors that are too small. However, overlap is most common for national-level estimates that include the same areas as sub-national data. After stratifying by level in **Table 2**, the effect of EVD on health care utilization remains significant. Overall, given the methodological limitations and presence of some bias, the estimates reported in this study should be taken as indications of the effect that EVD may have had on health services, rather than as precise estimates of that effect.

Finally, this review was not designed to decipher whether the decline in utilization observed was primarily due to supply or demand-side factors. This is important, since trust in health services is harder to rebuild than supply-side barriers (eg, staffing, infrastructure). We are also unable to quantify the impact of declining utilization on health outcomes such as morbidity or mortality. Declines in utilization may be due to less ill patients avoiding health facilities. Care not received at formal sector facilities may have been obtained from community-based or informal providers, who are a common source of care for childhood illness in West Africa [47]. However, declines were largest for non-elective procedures with no alternative source of care than formal sector facilities, such as C-sections, facility-based deliveries, and inpatient admissions. Therefore, the impact on health outcomes may have potentially been substantial.

## CONCLUSION

Beyond its direct death toll, the West African EVD outbreak significantly reduced health care utilization in the most-affected populations. The impact of this indirect effect on health and mortality from non-EVD causes, however, remains unknown. Given a large reduction in health service utilization over a large population for a period of up to 12 months, it has the potential to cause a number of additional deaths that might have exceeded Ebola’s direct impact. Estimates of this indirect toll will require modeling the consequences of the declines in health care utilization described in this paper, as well as retrospective mortality surveys (eg, Demographic and Health Surveys). In future outbreaks, the public health response must aim to maintain utilization of routine health services while also controlling the epidemic.
